# Prevalence and risk factors for low back pain among university teaching staff in Nairobi, Kenya: a cross-sectional study

**DOI:** 10.12688/f1000research.19384.1

**Published:** 2019-06-06

**Authors:** Saikou Yaya Kollet Diallo, Marshal Mutinda Mweu, Simeon Ochanda Mbuya, Mutuku Alexander Mwanthi

**Affiliations:** 1 Department of Public Health, Faculty of Health Sciences and Techniques, Gamal Abdel Nasser University of Conakry, Conakry, Guinea; 2 School of Public Health, College of Health Sciences, University of Nairobi, Nairobi, Kenya; 3 Department of Clinical Pharmacology, School of Medicine, College of Health Sciences, University of Nairobi, Nairobi, Kenya

**Keywords:** low back pain, prevalence, risk factors, University teaching staff.

## Abstract

**Background**: To date, there are few studies carried out on low back pain (LBP) among university teaching staff in developing countries despite academics being a high-risk group for LBP. In Kenya, to the best of our knowledge, there are no published studies that have investigated risk factors for LBP among teaching staff. The objectives of this study were to estimate the prevalence of LBP among teaching staff of the University of Nairobi (UoN), during the period June 2016 – May 2017, and to identify its socio-demographic and work-related risk factors.

**Methods**: An analytical cross-sectional study design was used to estimate the prevalence and investigate the risk factors for LBP among 136 teaching staff of UoN. A semi-structured questionnaire was used to collect data on LBP history, work-related and socio-demographic characteristics of the study participants. The 12-month prevalence of LBP and its associated 95% exact binomial confidence interval were estimated. A mixed-effects logistic regression model was used to evaluate the relationship between the predictors and LBP.

**Results**: The estimated 12-month prevalence of LBP was 64% (95% CI: 55.3%–72.0%). From the multivariable analysis, physical inactivity (aOR: 6.0; 95% CI: 1.2–29.6), office chairs without lumbar supports (aOR: 3.3; 95% CI: 0.1–0.9) and high workplace stress (aOR: 4.4; 95% CI: 1.1–17.5) were identified as significant risk factors for LBP among the respondents.

**Conclusions**: This study has revealed a high burden of LBP among teaching staff of the UoN and undoubtedly mimics the situation in other higher learning institutions in Kenya. Physical inactivity, sitting on chairs without lumbar supports and workplace stress have been identified as modifiable risk factors for LBP among teaching staff. This suggests a need to strengthen advocacy for regular physical activity, team-building activities and investment in office infrastructure to mitigate the effects of LBP within learning institutions.

## 1. Introduction

Disorders of the musculoskeletal system (MSDs) constitute the second most common cause of disability worldwide – accounting for 169,624,000 disability-adjusted life years (DALYs) as of 2010 which is a 45.5% increase over 10 years ^1, 2^. Of all work-related MSDs, low back pain (LBP) remains the most frequently diagnosed condition since the low back vertebral discs are subject to the greatest mechanical stress, compression force and degenerative changes ^3– 5^. LBP is defined as pain localised between the lower margin of the twelfth ribs and the lower gluteal folds with or without leg pain that lasts at least one day ^6, 7^.

LBP was ranked as the first contributing factor to global disability out of 291 conditions investigated in 2010 and the third in Eastern Sub-Saharan Africa, measured in years lived with disability ^7^. LBP prevalence was found to be 50% among physicians and dentists in India ^5^, 61.9% among Ugandan nurses ^4^ and 77.2% in theatre nurses in Nigeria ^3^. It is estimated that more than 80% of people end up suffering from LBP at some point in their lifetimes ^8^. Only 5–15% of LBP cases have a specific cause such as an osteoporotic fracture, neoplasm or infection ^9^.

LBP arises from several contributing factors, namely: socio-demographic, ergonomic and psychosocial predictors ^10^. Low back injuries leading to LBP are associated with occupational risk factors, with 11% to 80% of them being attributable to ergonomic factors such as prolonged sitting, lifting, bending and twisting ^10– 13^. Psychosocial factors account for 14% to 63% of low back injuries, mainly high job demands, job dissatisfaction and stress at the workplace ^10– 12, 14^. Socio-demographic factors equally play an important role in LBP occurrence and comprise both individual and lifestyle factors. Of these, the most commonly identified are lack of physical exercise, old age, female gender, obesity and smoking ^11– 15^.

The job description for teachers comprises a broad range of duties and responsibilities which may predispose teachers to LBP. For instance, while preparing teaching materials, teachers may experience prolonged sitting either in the office or at home. When delivering lectures, they may be upstanding for long hours, or may adopt awkward postures like bending, reaching and twisting. They may have to use inappropriate furniture such as immobile chairs without back support and non-mechanized tables. These varying postures may trigger back pain owing to the continuous loading of back muscles ^12, 16, 17^. In Kenya, little has been published on LBP. However, the few available studies showed high prevalence of the condition in Nairobi: 76.5% among sedentary office workers ^18^ and 90.5% among hospital employees ^19^. With a considerable proportion of teaching staff in Kenya being past the age of 50 years, it is anticipated that the magnitude of LBP would be high with attendant productivity losses and financial burden to the University community.

The objectives of this study were to estimate the prevalence of LBP among the teaching staff of the College of Health Sciences, University of Nairobi, during the period June 2016-May 2017, and to identify its socio-demographic and work-related risk factors with a view to informing the formulation of effective prevention and control strategies for LBP within teaching institutions in Kenya.

## 2. Methods

### 2.1 Study area and design

The study was conducted at the University of Nairobi (UoN), College of Health Sciences (CHS) – one of the six constituent colleges of the UoN. Notably, UoN is the largest higher learning institution in Kenya, whose working conditions closely mimic those of other public tertiary institutions in the country. The CHS consists of five schools (Medicine [SOM], Dental Sciences [SDS], Pharmacy [SOPharm], Nursing Sciences [SON], and Public Health [SPH]) and four institutes (Tropical and Infectious Diseases [UNITID], Kenya AIDS Vaccine Initiative [KAVI], East African Kidney Institute [EAKI] and the Centre for HIV Prevention and Research [CHIVPR]).

An analytical cross-sectional study design was employed to estimate the prevalence and investigate the risk factors for LBP among the college teaching staff of UoN from June 2016 to May 2017. The study was reported as per the STROBE guidelines for reporting observational studies ^20^.

### 2.2 Study population, eligibility and selection of participants

**Figure 1.**
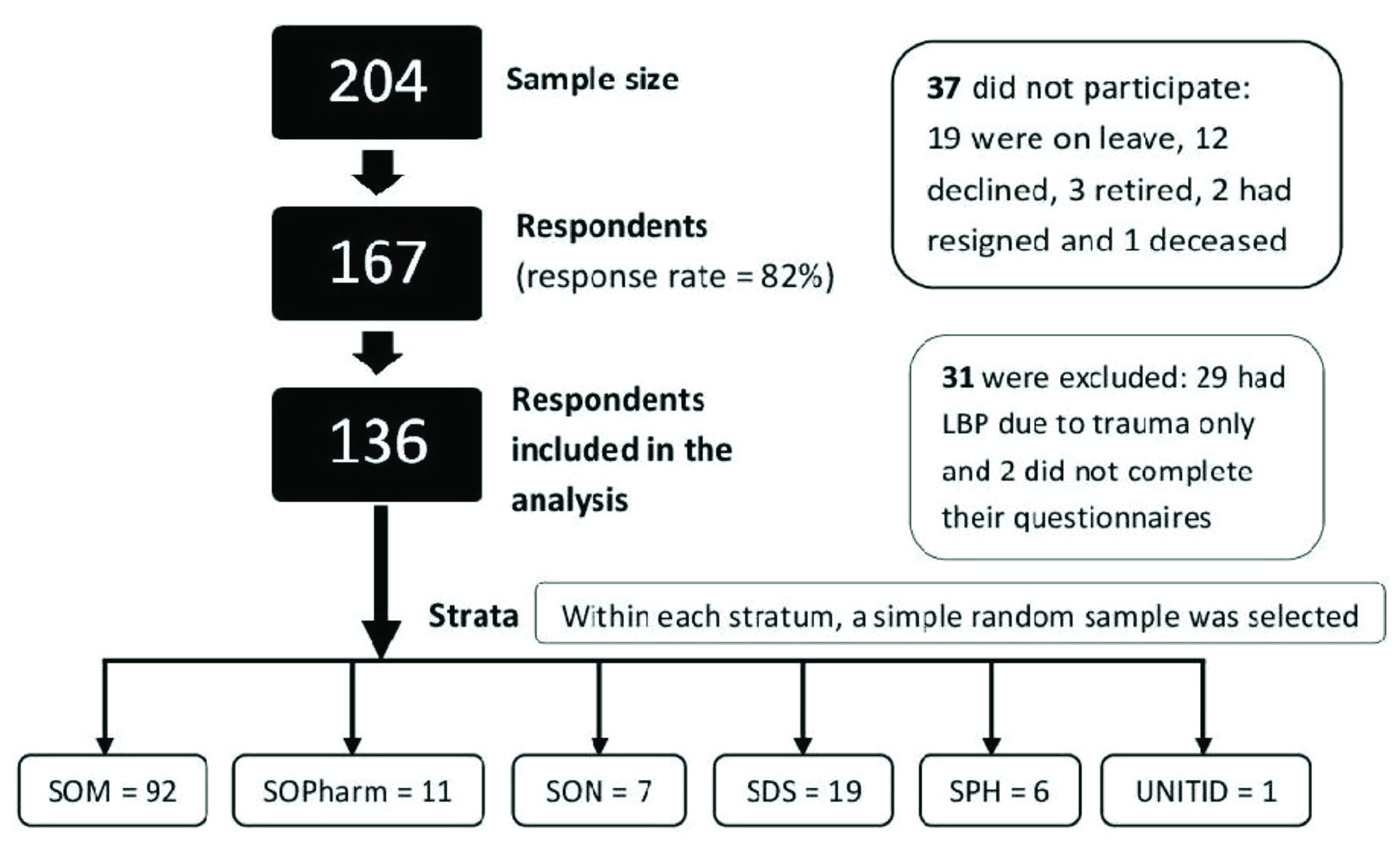
Flow chart of the stratified random sampling strategy.

The study population consisted of all teaching staff of the CHS eligible to participate in the study. To be eligible for participation, a staff member had to have been employed for at least 12 months prior to the commencement date of the study (May 2017) and have given informed written consent for participation. Moreover, those having LBP due to trauma, infection or tumour were excluded from the study. The sampling frame of teaching staff was secured from the college registry. To obtain the study sample, a stratified random sampling technique (with strata being the constituent Schools and Institutes of the CHS) was used. Within each stratum, a simple random sample was selected, such that the number sampled per stratum was proportional to the size of the stratum. Arguably, stratified random sampling ensures that all strata are represented in the sample and further improves precision of the estimates by removing the between-strata variation ^21^. A flow chart of the sampling strategy is shown in Figure 1.

### 2.3 Outcome definition

An LBP case was defined as a staff member who had a history of pain localised in the lower back (as previously defined) lasting for at least 24 hours within the 12-month study period, had been physically examined by a physician at a health facility and further undergone a diagnostic imaging examination revealing lumbar disc degeneration. Contrastingly, a non-case was a study participant without a previous history of LBP within the same study period.

### 2.4 Sample size determination

The required sample size was determined as specified by Kelsey, JL *et al*. ^22^ for cross-sectional studies:

$$\begin{array}{l} n_{1}=\frac{{\left(Z_{\alpha }+Z_{\beta }\right)}^{2}\bar{p}\bar{q}(r+1)}{r{\left(p_{1}-p_{2}\right)}^{2}}\\ \;\bar{q}=1-\bar{p}\;n_{2}=rn_{1} \end{array}$$

$$p_{1}=\frac{p_{2}OR}{1+p_{2}(OR-1)}$$

$$\bar{p}=\frac{p_{1}+\text{r}p_{\text{2}}}{r+1}$$

Where:

*n*_1_ is the number of cases and *n*_2_ is the number of non-cases; *p*_1_ is the proportion of individuals who did not exercise and had LBP; *p*_2_ is the proportion of individuals who exercised and had LBP – estimated to be 43.1% based on a previous study ^14^. Notably, *Z _α_*_/2_ (1.96) and *Z _β_* (-0.84) are the values which specify the desired 2-tailed confidence level (95%) and statistical power (80%) respectively. The odds ratio ( *OR*) for the effect of the primary exposure (lack of physical exercise) was hypothesised to be 2.2 ^14^. The ratio ( *r*) of unexposed to exposed individuals was set at 1. Given these figures, a total sample size of 204 participants was derived.

### 2.5 Data collection and study variables

**Figure 2.**
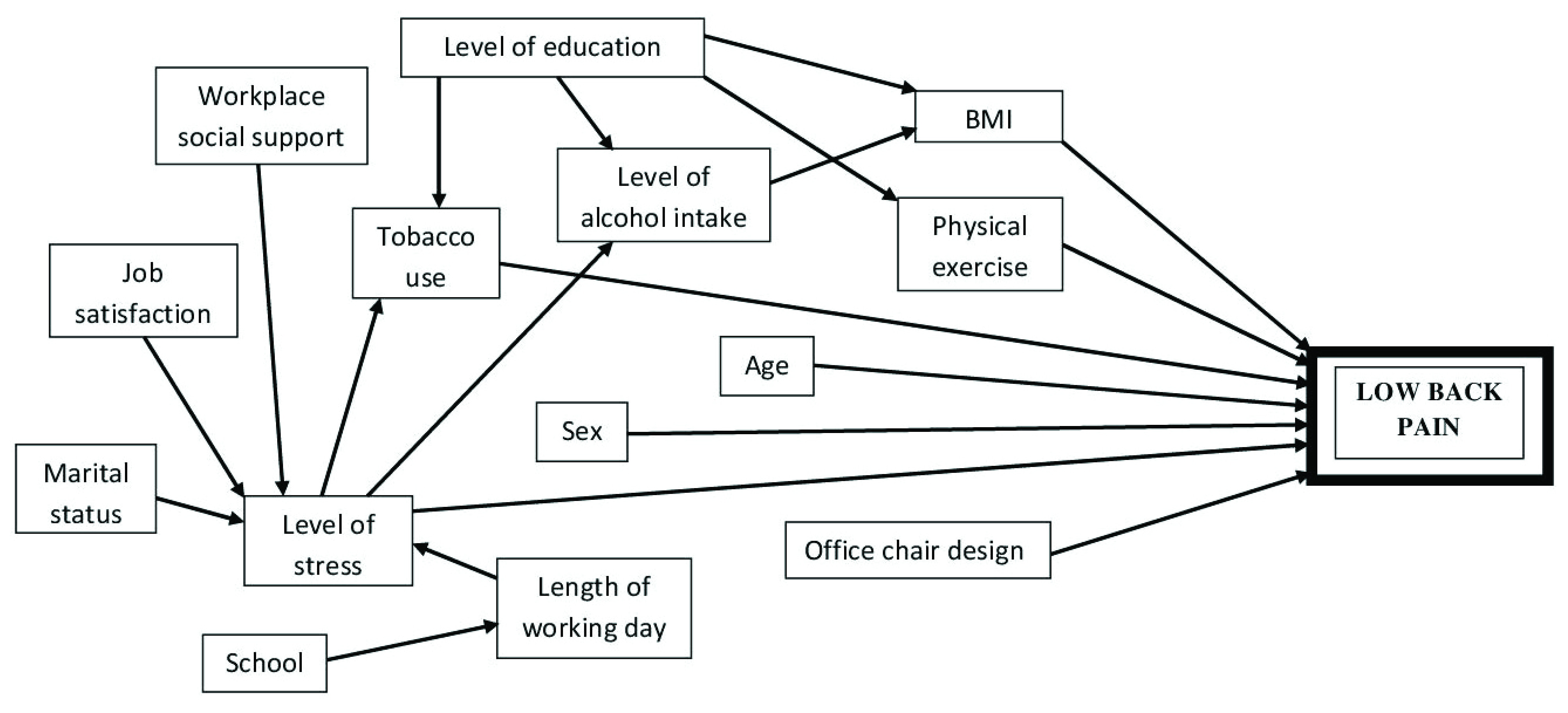
Causal diagram of factors thought to influence Low back pain occurrence among teaching staff of the College of Health Sciences, University of Nairobi.

Initially, two research assistants were recruited and trained to aid with the data collection exercise that spanned a two-month period May 31 ^st^–July 31 ^st^ 2017. As for the data collection, a semi-structured questionnaire (see extended data ^23^) was administered to the study participants capturing details of their LBP history, and predictors: work-related (length of working day, office chair design, stress, social support and job satisfaction) and socio-demographic characteristics (age, sex, marital status, body mass index (BMI), level of education, school (including department), physical activity engagement, tobacco use and level of alcohol intake). The predictors were assessed as given in Table 1. A conceptual framework depicting the predictor-outcome relationship is displayed in Figure 2.

**Table 1.** Predictor variables and their measurements.

Variable (type)	Measurement
Age (continuous)	Captured in years.
Sex (nominal)	Entered as male or female.
Marital status (nominal)	Assessed in three levels: single, married or others (widowed, divorced and separated).
BMI (continuous)	The body mass index (BMI) was determined by dividing weight in kilogrammes by height in metres squared.
School (nominal)	The institutional entity of the CHS where the teaching staff is based (including the specific department). Grouped into five levels: SOM (+UNITID & EAKI), SOPharm, SON, SDS, SPH (+CHIVPR).
Level of education (ordinal)	The level of university training attained by the teaching staff. Assessed in three levels: Bachelors, Masters or PhD.
Physical exercise (ordinal)	Physical exercise entails engaging in any of the following activities by the teaching staff: walking, running, cycling, swimming, jogging, back exercise and playing games e.g. football. This was graded in three levels according to the duration of continuous activity per day and frequency per week ^24^: grade 1 or never (frequency less than once a week); grade 2/rare (1 or 2 days per week for a minimum of 30 minutes each day); grade 3/regular (at least 3 days per week for a minimum of 30 min each day).
Tobacco use (nominal)	Either by smoking or chewing and assessed either as user or non-user
Level of alcohol intake (ordinal)	This represents the amount of alcohol that is consumed by the teaching staff per week. Classified into three categories based on the frequency of intake per week ^24^: grade 1 or non-consumer (less than once a week); grade 2/rare consumer (1-3 times in a week); grade 3/regular consumer (4-7 times per week).
Length of working day (continuous)	This constitutes the time during which the teaching staff is performing work-related duties.
Office chair design (nominal)	Assessed in two levels: with or without lumbar support.
Level of workplace stress (ordinal)	This refers to an uncomfortable feeling of nervousness or great worry caused by any difficult situation related to one’s work. Therefore, a stressor may be any physical or psychological threat to safety, status, or well-being; physical or psychological demands that exceed available resources; any unpredictable change in the work environment; or any inconsistency between expectations and outcomes. It was scaled into three levels: 1 = low; 2 = medium; 3 = high.
Workplace social support (ordinal)	The degree to which the teaching staff perceives that his/her well-being is valued by his colleagues (can be in form of material, emotional or informational support). It was categorized into four levels: 0 = absent, 1 = poor, 2 = ok/satisfactory or 3 = good.
Job satisfaction (ordinal)	The feeling of pleasure and achievement that the teaching staff experiences in his/her job when he/she knows that his/her work is worth doing, or the degree to which his/her work gives him/her this feeling. It was categorized into three levels: 1 = dissatisfied, 2 = neutral or 3 = satisfied.

### 2.6 Ethical considerations

The study respondents provided written informed consent expressing their willingness to take part in the study. Approval for the study was granted by the Kenyatta National Hospital and University of Nairobi joint Ethics and Research Committee (KNH-ERC/A/171).

### 2.7 Minimisation of biases

Granted that cross-sectional studies are prone to a range of biases that may invalidate study results, deliberate attempts were made to minimize their occurrence. Ergonomic and lifestyle factors are readily modified once individuals are diagnosed with LBP. As such, to reduce the possibility of reverse causality involving these set of factors, specific questions targeted the period preceding the onset of symptoms characteristic of LBP for case respondents. To standardise the interview process and thus minimize interviewer bias, the research assistants were trained on sound interviewing techniques. As non-response may introduce selection bias in cross-sectional studies, non-responders were aggressively followed up with reminders to achieve a reasonable response rate.

### 2.8 Data processing and statistical analysis

Prior to data entry, questionnaire responses capturing qualitative variables were coded. The data were then double-entered in an EpiData v3.1 spreadsheet by two independent data entry clerks to minimize errors. The validated dataset was then exported to Stata v13 software for data cleaning and analyses. Continuous variables were summarized using the median and inter-quartile range (IQR) as well as histograms and boxplots. For categorical variables, proportions were computed. The prevalence of LBP and its associated 95% exact binomial confidence interval were estimated. Code for analysis is available as extended data ^25^.

For univariable analyses, a mixed-effects logistic regression model was used to evaluate the effect of each predictor on LBP, with the variable *department* included as a random effect to account for clustering of the outcome within departments. The significance of each of the predictors at this stage was evaluated at a liberal *P*≤0.20. As inclusion of *age*, *BMI* and *length of working day* as continuous predictors in the univariable models yielded insignificant results, these were categorised and reassessed for significance. In particular, *age* was grouped into three categories: ≤43yrs; 44–57yrs; ≥58yrs, *BMI* was classified into the four BMI categories ^26^: Underweight(<18.5), Normal weight (18.5–24.9), Overweight (25.0–29.9) or Obese (≥ 30.0) and *length of working day* was categorised into: ≤8hrs or >8hrs.

Variables that were found to be significant in the univariable analyses were then offered to a multivariable model where a backward step-wise approach was used to eliminate variables at *P*≥0.05. To minimize confounding, elimination of non-significant predictors was only considered when their exclusion from the model did not result in a more than 30% change in the effects of the remaining variables ^21^. Two-way interactions were fitted between the remaining variables of the final model and assessed for significance.

## 3. Results

### 3.1 Descriptive statistics

A total of 204 teaching staff of the CHS were invited to participate in the study, from whom 167 consented to participating in the survey, giving a response rate of 81.9%. However, of the 167 participants, 31 were excluded from the analyses for reporting trauma and/or infection as the reason(s) for their back pain. Therefore, 136 participants were considered in the analyses [see underlying data].

Descriptive statistics for the predictors of LBP are displayed in Table 2. Notably, the median age for the participants was 51 years (Range: 31–81yrs). A typical working day was 10 hours long (range: 4–18hrs). Only 44.9% (n=61) of the participants regularly exercised. Participants with office chairs that had lumbar support represented 41.9% (n=57) of the total. The estimated 12-month period prevalence of LBP was 64% (95% CI: 55.3%–72.0%). 

**Table 2.** Descriptive statistics for the predictors of low back pain among teaching staff of the College of Health Sciences, University of Nairobi, Kenya (n=136).

Variable	Values	Median	Inter-quartile range (IQR)	Frequency n (%)
Sex	Male Female	- -		86 (63.2) 50 (36.8)
Age (years)	31.0 – 81.0	51.0	18.5	-
Marital status	Married Single Others	- - -		116 (85.3) 13 (9.6) 7 (5.2)
BMI (Kg/m ^2^)	16.7 - 38.1	28.0	4.9	-
School	SOM SOPharm SON SDS SPH	- - - - -		93 (68.4) 11 (8.1) 7 (5.2) 19 (14.0) 6 (4.4)
Level of education	Bachelors Masters PhD	- - -		6 (4.4) 94 (69.1) 36 (26.5)
Physical exercise	Never Rarely Regularly	- - -		29 (21.3) 46 (33.8) 61 (44.9)
Tobacco use	Non-user User	- -		133 (97.8) 03 (2.2)
Level of alcohol intake	Non-consumer Rare Regular	- - -		88 (64.7) 42 (30.9) 6 (4.4)
Office chair design	Without LS With LS	- -		79 (58.1) 57 (41.9)
Length of working day	4.0 – 18.0	10.0	3.5	-
Level of workplace stress	Low Medium High	- - -		46 (33.8) 49 (36.0) 41 (30.2)
Workplace social support	Absent Poor Satisfactory Good	- - - -		32 (23.5) 35 (25.7) 58 (42.7) 11 (8.1)
Job satisfaction	Dissatisfied Neutral Satisfied	- - -		17 (12.5) 62 (45.6) 57 (41.9)

### 3.2 Logistic regression analyses

Based on results of the univariable analyses, the variables: *sex, age, school*, *physical exercise, office chair design* and *level of workplace stress*, were significantly associated with LBP at *P* 0.20 ( Table 3). These were subsequently offered to the multivariable model. In the multivariable analysis, only *physical exercise*, *office chair design* and *level of workplace stress* were shown to be significant predictors of LBP at the 5% of significance level ( Table 4).

**Table 3.** Univariable analysis of the risk factors for LBP among teaching staff of the College of Health Sciences, University of Nairobi, Kenya, using mixed-effects logistic regression with the variable *department* included as a random effect.

Variable	Values	LBP - (n=49)	LBP+ (n=87)	OR	95%CI		LRT *P*-value
		n	n		Lower	Upper	
Sex ^a^	Male Female	35 14	51 36	1.0 2.0	- 0.9 – 4.5		0.098
Age (years) ^b^	31-43 44-57 58-81	16 11 22	30 34 23	1.0 1.8 0.6	- 0.7 – 4.7 0.2 – 1.4		0.048
Marital status	Married Single Others	44 02 03	72 11 04	1.0 3.3 0.8	- 0.7 – 16.0 0.2 – 3.9		0.234
BMI (in Kg/m ^2^)	Normal-weight Over-weight Obese	10 24 15	22 43 22	1.2 1.0 0.8	0.5 – 3.2 - 0.4 – 1.9		0.720
School ^c^	SOM SOPharm SON SDS SPH	33 01 02 09 04	60 10 05 10 02	1.0 5.5 1.4 0.6 0.3	- 0.7 – 44.9 0.3 – 7.5 0.2 – 1.7 0.0 – 1.6		0.109
Level of education	Bachelors Masters PhD	01 35 13	05 59 23	2.9 1.0 1.1	0.3 – 27.3 - 0.5 – 2.4		0.585
Physical exercise ^d^	Never Rarely Regularly	03 14 32	26 32 29	13.3 3.3 1.0	3.2 – 55.4 1.3 – 8.8 -		0.000
Tobacco use	Non-user User	47 02	86 01	1.0 0.3	- 0.0 – 3.5		0.318
Level of alcohol intake	Non-consumer Rare consumer Regular consumer	31 14 04	57 28 02	1.0 1.1 0.3	- 0.5 – 2.4 0.0 – 1.7		0.324
Office chair design ^e^	Without LS With LS	17 32	62 25	1.0 0.2	- 0.1 – 0.4		0.000
Length of Working day	≤ 8 > 8	09 40	22 65	1.6 1.0	0.6 – 4.1 -		0.301
Level of workplace stress ^f^	Low Medium High	26 17 06	20 32 35	0.3 1.0 3.9	0.1 – 0.9 - 1.2 – 13.0		0.000
Workplace social support	Absent Poor Satisfactory Good	13 11 21 04	19 24 37 07	0.8 1.3 1.0 0.9	0.3 – 2.0 0.5 – 3.2 - 0.2 – 3.8		0.833
Job satisfaction	Dissatisfied Neutral Satisfied	06 18 25	11 44 32	0.7 1.0 0.5	0.2 – 2.4 - 0.2 – 1.1		0.264

^a, b, c, d, e, f ^Variables eligible for inclusion in the multivariable model (P≤0.20)

**Table 4.** Multivariable analysis of the risk factors for LBP among teaching staff of the College of Health Sciences, University of Nairobi, Kenya, using mixed-effects logistic regression with the variable *department* included as a random effect.

Variable	Values	aOR ^a^	95% CI		LRT *P*-value
			Lower	Upper	
Physical exercise	Never participate Rarely participate Regularly participate	6.0 2.8 1.0	1.2 – 29.6 0.9 – 8.4 -		0.031
Office chair design	Without lumbar support With lumbar support	1.0 0.3	- 0.1 – 0.9		0.021
Level of workplace stress	Low Medium High	0.6 1.0 4.4	0.2 – 1.9 - 1.1 – 17.5		0.011

^a^Adjusted odds ratio

Compared to respondents who regularly exercised, participants who rarely and never exercised had respectively about three (aOR: 2.8; 95% CI: 0.9–8.4) and six times (aOR: 6.0; 95% CI: 1.2–29.6) the odds of LBP controlling for their office chair design and workplace stress level. Participants who sat on chairs with lumbar support had a third (aOR: 0.3; 95% CI: 0.1–0.9) the odds of LBP as those who did not regardless of their level of physical activity and stress at their workplace. Irrespective of their level of physical activity and design of their office chair, respondents who experienced high and low stress levels at their workplace had roughly four times (aOR: 4.4; 95% CI: 1.1–17.5) and three-fifths (aOR: 0.6; 95% CI: 0.2–1.9) the odds of LBP respectively, as those whose perception of stress was medium.

## 4. Discussion

### 4.1 Prevalence of LBP

The prevalence of LBP among teaching staff of the CHS, UoN was estimated to be 64.0%. This is a higher prevalence than demonstrated by most studies conducted among teachers in which LBP prevalence ranged between 22.3% (Thailand) and 57.5% (Ethiopia) ^12– 15^. This variation could be attributable to age differences between the study participants, with those in the mentioned studies being on average younger (mean age: 34.7–38yrs) than those included in the present study (mean age: 50.9yrs). An age-LBP association has been demonstrated, with LBP being more prevalent among individuals over 40 years ^12, 14, 27^.

### 4.2 Risk factors for LBP

This study has shown that teachers who either do not exercise or do so infrequently, have higher odds of experiencing LBP than their counterparts who exercise regularly. This finding is consistent with study observations made in Israel, Iran, India, South Korea and Ethiopia ^11, 14, 15^. Regular physical exercise has been shown to strengthen lower back muscles and maintain the spine in proper alignment for optimal function. Furthermore, routine exercises increase blood supply to the spine muscles, joints and intervertebral discs minimizing injury and enhancing their repair ^14, 28, 29^. It has been suggested that a minimum of 30 minutes of regular exercise could increase trunk flexibility and stimulate an adequate production of endorphins that could diminish pain sensation ^30, 31^.

Sitting on a chair with back support had the effect of lowering the odds of LBP. The use of lumbar supports has been widely advocated because of their well-known function of preserving the integrity of the low back curves, thus reducing the risk of LBP ^32– 34^. Additionally, the tilt of the lumbar support permits the person using it to sit with his/her upper body slightly reclined which ensures proper body weight distribution ^32, 35, 36^.

There was a noticeable association between perceived stress levels at the workplace and the reporting of LBP. High stress levels have been associated with the stimulation of the sympathetic nervous system prompting the release of stress mediators that can strain the musculoskeletal system resulting in LBP. Our finding concurs with that reported by an Ethiopian study in which participants reporting stress had roughly two times the odds of experiencing LBP than those without stress ^14^. Similar associations have also been observed elsewhere ^11, 28^.

Our study did not suggest any evidence for the existence of a real difference in LBP prevalence between sexes. This could be partly ascribable to our study participants being generally older and hence exposed to a similar risk. Nevertheless, among younger participants, being female has been associated with an elevated risk of LBP owing to hormonal imbalances ^11, 14, 37^. More so, during pregnancy, hormonal changes responsible for loosening the spinal ligaments coupled with the extra weight that stresses the lower back muscles heighten the risk of LBP ^38, 39^.

Taking into account other study variables, age did not emerge as a significant predictor for LBP in the present study. A likely explanation for this would be that older participants aware of their disproportionately higher risk, engaged themselves in regular exercises at a comparably higher frequency (as per the data: *P*=0.02) thus arguably, balancing out their LBP risk to that of their younger counterparts. Nonetheless, a number of studies have reported age as a significant risk factor for LBP; old age being associated with spine and vertebral disc degeneration as well as loss of connective tissue elasticity that can result in LBP ^12, 14, 29^.

Working at a particular school did not significantly influence a participant’s likelihood of LBP. This is conceivable considering that the respondents are likely to have similar work responsibilities entailing preparation of teaching materials, lecturing, grading of students’ papers, mentoring and supervision that often demand extended periods of sitting and standing. This prevalence homogeneity may also denote uniformity in the distribution of office furniture designs across schools.

A couple of limitations are inherent in the present study. Definition of the outcome was pegged on self-report which could have introduced non-differential misclassification with a potential to bias the estimated odds ratios towards null. As measurement of exposures relied on recall which could have been incomplete especially for chronic cases, this would have the potential of biasing the effect estimates. Since non-responders tend to systematically differ from responders with regards to a range of health outcomes, it is anticipated that the current study’s prevalence underestimates the true burden of LBP in this study population. In light of the above-mentioned limitations, it should be borne in mind that the results of this study are merely hypothesis generators and hence, stronger study designs such as case-control or cohort studies are recommended to validate the findings.

## 5. Conclusions

This study has revealed a high burden of LBP among teaching staff of the University of Nairobi and undoubtedly mimics the situation in other higher learning institutions in Kenya. Lack of physical activity, seating on chairs without lumbar support and workplace stress have been identified as modifiable risk factors for LBP among teaching staff. Considering the similarity in demographics and working conditions across public institutions in Kenya, these findings are readily generalisable to other public tertiary institutions within the country. Consequently, there is a pressing need for university managements to: (1) invest in suitable office furniture, in particular, office chairs fitted with appropriate lumbar supports and (2) raise advocacy for and facilitate the implementation of regular workouts and departmental team-building activities with a view to mitigating the burden of LBP among their staff.

## 6. Data availability

### Underlying data

The raw dataset for the study is kept under restricted access since it contains sensitive participant information. Access to the raw data is possible upon placing a formal request to the corresponding author ( disykgn@gmail.com). The replication data, analysis script and questionnaire for this manuscript are available from figshare.

Figshare: LBP_UoN_CHS_2.dta. https://doi.org/10.6084/m9.figshare.8148779.v1 ^40^

This project contains the following underlying data:

- LBP_UoN_CHS_2.dta (Low back pain survey data)

Data are available under the terms of the Creative Commons Zero "No rights reserved" data waiver (CC0 1.0 Public domain dedication). 

### Extended data

Figshare: LBP Study Questionnaire_UoN. https://doi.org/10.6084/m9.figshare.8197598.v1 ^23^

This project contains the following extended data:

- Questionnqire_LBP Study among teaching staff UoN, Kenya.pdf (Low back pain survey questionnaire)

Data are available under the terms of the Creative Commons Zero "No rights reserved" data waiver (CC0 1.0 Public domain dedication).

Figshare: LBP study_CHS_UoN_Stata code. https://doi.org/10.6084/m9.figshare.8197715.v2 ^25^

This project contains the following extended data:

- LBP study_Kenya_UoN_CHS_Stata Code.do (Low back pain Stata code)

Data are available under the terms of the MIT licence.
