# Supplementary material for: Prevalence and risk factors for low back pain among university teaching staff in Nairobi, Kenya: a cross-sectional study
Source: F1000Res. 2019 Jun 6;8:808. [Version 1] doi: 10.12688/f1000research.19384.1 (PMC7531051; doi:10.12688/f1000research.19384.1)
Supplement: Peer review history

# Peer review: Reviewer response for version 1

**DOI:** 10.5256/f1000research.21250.r70536

**Published:** 2020-10-01

**Authors:** Oyelola A Adegboye

**Affiliations:** 1 Australian Institute of Tropical Health and Medicine, James Cook University, Townsville, Qld, Australia

My comments  majorly focused on the methods of this study:

1. One of the major weakness of this study is the use of “type of furniture” as a measure of sedentary behavior, this is not a sufficient risk factor. It is unclear to me why Q16 (hours of sitting) of the questionnaire was not actually explored together with the type of chair? The authors should at least acknowledge the lack of measurable factors for sedentary behavior, such as hours of sitting in their limitation. Additionally, there are several validated and standardized questionnaires for measuring some of the risk factors associated with LBP and sedentary, I am surprised that the authors did not explore this option. What is the reliability and validity of the test instrument used in this study?
2. Why was the study restricted to one college? In addition to teaching, I am assuming these staff also engaged in medical practices such as ward rounds and attending to patients, therefore it cannot be generalized to university teaching staff as the title suggests.
3. Rather than saying the significance of variables were evaluated at p≤0.2, they could have said “…predictors with p≤0.2 in the univariable analysis were included in the multivariable  analysis.”
4. The authors sampled exactly 204 does not make sense, the authors should have invited an additional 10% participants to account for possible refusal as we have seen in this study, an actual sample size of 167 instead of the proposed 204 (80% power).

Is the work clearly and accurately presented and does it cite the current literature?

Partly

If applicable, is the statistical analysis and its interpretation appropriate?

Yes

Are all the source data underlying the results available to ensure full reproducibility?

No

Is the study design appropriate and is the work technically sound?

Partly

Are the conclusions drawn adequately supported by the results?

Partly

Are sufficient details of methods and analysis provided to allow replication by others?

Yes

Reviewer Expertise:

Biostatistics, exposure science, multi-level modelling

I confirm that I have read this submission and believe that I have an appropriate level of expertise to confirm that it is of an acceptable scientific standard, however I have significant reservations, as outlined above.

# Peer review: Reviewer response for version 1

**DOI:** 10.5256/f1000research.21250.r58991

**Published:** 2020-03-02

**Authors:** Peter R. Croft

**Affiliations:** 1 Arthritis Research UK Primary Care Centre, Research Institute for Primary Care and Health Sciences, Keele University, Keele, UK

It is good to see original studies on low back pain (LBP) from Africa. LBP is a global problem and yet is under-represented in research from countries such as Kenya.

 This is a generally well-written paper, with appropriate methods and analysis and presentation of results. So the overall opinion is that this is a useful, well-performed and reasonably well-reported study.  

 There are some weaknesses however, and they are discussed below in the hope that they will help interpretation of results and be constructive for future research by the authors.

**1. Study rationale:**

 The authors carefully chose a narrow focus for their paper. This study is about LBP in University teaching staff, and the limit of the generalisations made at the end of the paper is that results can be extended to teaching staff in other Higher Education institutions in Kenya. The authors’ main conclusions relate to a proposal that the risk factors for low back pain identified in the study should and could be reduced in these workplaces.

 This is admirable as a stimulus to local preventive action, but the interest for an international readership would be (a) to consider to what extent the prevalence estimate in this workplace population represents the occurrence of LBP in older Kenyans and Africans more widely, and (b) to know how the findings on the risk factors for LBP compare with and contribute to the extensive global literature on these factors. Such wider objectives would draw out the study’s importance beyond the local occupational setting, but require a more detailed discussion section (see below).

**2. Study context:**

 It is rather alarming to read that this observational study of LBP in a random sample of teachers in a University health faculty could take as its case definition *low back pain of minimum 24 hours plus visit to health care plus spinal imaging*. Given that international guidelines recommend strongly against X-ray for uncomplicated back pain, does this mean that in Kenya most people experiencing back pain will expect to have an X-ray (which would be important contextual information for the paper) OR does it mean that this is a highly selected sample of all those in this population who had LBP (i.e. were there many people reporting back pain who had not had an X-ray and were therefore excluded from the study? – this would be important for study interpretation).

**3. Study style:**

 The authors have appropriately and importantly included workplace stress and supervisor support and demographic/lifestyle factors among the potential risk factors for LBP. This is excellent, but much of the introduction and discussion defaults to “physical” explanations of, and mechanisms for, LBP. Chronic LBP is a complex biopsychosocial phenomenon, in which mental health and cultural perceptions play as big a role as mechanical factors, and this could be recognised more explicitly in the introduction and discussion sections.

**4. The definition of low back pain:**

 The definition of the area of the back to be included is admirably clear, as is the minimum duration of 24 hours. However it is unfortunate (especially for the estimate of prevalence) that there were no further details on the pain itself and its impact, in order to sub-group the prevalence estimate according to severity. Back pain is so common that, to provide prevalence estimates that are more widely useful for planning, prevention and care, it should be standard practice in prevalence surveys to add a measure of severity and impact, such as that in Von Korff et al, Pain, 2020. ^1^ (The authors might argue that this was not so necessary for the risk factor component of their study but that is one reason for clearly separating the discussion of prevalence and risk factor components of the study – see comments below).

**5. Study design:**

 The authors say at the end of their paper that more research with stronger designs (including case-control) is needed to confirm the results found in their cross-sectional study. I think the authors are underplaying their own design. There is an argument for saying that their study has two components – first, a cross-sectional population survey to estimate prevalence, and second, a population-based case-control study to study associations with LBP.

 The arguments for suggesting that the risk factor study is a case-control study are:

1. Exposures were compared between those without LBP in the survey who represent a population sample of “controls”, and “cases” with LBP (after exclusion of cases with other causes (injury/infection)).
2. The sample size has been estimated on the basis of exposure data, as for a case-control study, rather than for the prevalence study.
3. The authors have attempted to ensure that, when gathering exposure data, participants were asked to recall exposures in periods prior to the onset of their LBP – a classic piece of case-control design.

 

 If these two components of their study were separated, then the authors could usefully critically reflect on the following design points:

 a. It was good to see a flow diagram of response and exclusion, and a high response rate, for the prevalence study. But what was the size of the total eligible population that provided the basis for stratified sampling for the study population (before exclusions, invitations, and nonresponse)?

 A small point to note is that the word “aggressive” in relation to following up non-responders is best avoided. It is better to give the details of exactly what was done, including a clear account of how and when people’s non-response is taken as final.

 b. What was the origin of the questions used in the semi-structured interviews, and had there been any investigation of questionnaire validity prior to the main study? For example, alternative external records on the “lumbar support” seating data might have been sought from departmental purchasing data; repeatability of physical activity recall might have been tested by repeat interviews in a sub-sample of participants; were the interviewers aware of the participants’ back pain status when they asked about the risk exposures? Even if these “safeguards” were not carried out, the authors could consider adding a critical discussion about what they might have added to the credibility and validity of the study.

**6, Study analysis:**

1. Independent data entry is excellent practice – but some details of how any disagreements were handled would be useful, together with a summary of such disagreements.
2. Medians are fine but it might be better to make a statement about distributions of the relevant continuous variables because looking at them it is unclear why means and standard deviations were not chosen. The authors seem a little uncertain themselves because mean age is quoted in the discussion and median age in methods and results.
3. The analysis of results was nicely structured and followed a clear and appropriate pathway from descriptive to univariable to backwards multivariable models. However there was often a slightly ‘formulaic’ style to the writing of this, which could be avoided and softened by inclusion of some actual descriptive data in the text.
4. Mention should be made that the high prevalence of some exposures means that the odds ratios are likely to be overestimates of actual risk.

**7. Discussion:**

 This was the weakest part of the paper and could have been a lot stronger.

1. The section on bias again seemed rather ‘textbook’ in style and content, and does not convey the sense of a rigorous critical discussion of the specific issues raised by this study. More detail from the study itself and more detailed discussion of the individual points would help.
2. The authors raise as a limitation that the outcomes (presumably meaning back pain present or absent) are based on self-report – but it is difficult to see how the presence of back pain could be based on anything else but self-report. So this should rather have been a discussion of what the effects of misclassification of cases and controls might have been.
3. The sentence that states *“it is anticipated that the current study’s prevalence underestimates the true burden of LBP in this study population”* is decidedly odd, given the initial high response rate. This is where it would help to link the discussion back to the rationale for the study (what population is the prevalence trying to represent? How much bias could an initial 20% response possibly introduce?).
4. The final sentence of the discussion is not convincing.I don’t think it is right to say this is a “hypothesis generating” study – it does not fit with their main conclusion which urges all educational institutions in Kenya to look at how to modify risk factors in the workplace. This is an important conclusion but needs to be reached from (a) careful critical assessment of whether there study has established ‘cause’ or just association, and (b) rigorous comparison of their results with the wide international literature on these risk factors to see if their study is consistent with that literature and therefore strong enough to drive recommendations.
5. Finally there should be a separate conclusion about the prevalence study, with suggestions of how the authors’ methodology might be used as the basis for more research to establish prevalence in other sectors of Kenyan society and why this might be useful to know. The authors are helpfully comprehensive about how their study fits with other prevalence studies in Africa – this is so important - but they could perhaps acknowledge that severity and impact must be added to future prevalence studies in their country.

Is the work clearly and accurately presented and does it cite the current literature?

Partly

If applicable, is the statistical analysis and its interpretation appropriate?

Partly

Are all the source data underlying the results available to ensure full reproducibility?

Yes

Is the study design appropriate and is the work technically sound?

Partly

Are the conclusions drawn adequately supported by the results?

Partly

Are sufficient details of methods and analysis provided to allow replication by others?

Yes

Reviewer Expertise:

Epidemiology

I confirm that I have read this submission and believe that I have an appropriate level of expertise to confirm that it is of an acceptable scientific standard, however I have significant reservations, as outlined above.

## References

[1] : Graded chronic pain scale revised: mild, bothersome, and high-impact chronic pain. *Pain*.2020;161(3) : 10.1097/j.pain.0000000000001758 651-661 10.1097/j.pain.0000000000001758 31764390PMC7097879

# Peer review: Reviewer response for version 1

**DOI:** 10.5256/f1000research.21250.r59230

**Published:** 2020-02-24

**Authors:** Marcus Fraga Vieira

**Affiliations:** 1 Bioengineering and Biomechanics Laboratory, Federal University of Goiás, Goiânia, Brazil

This manuscript deals with the prevalence of low back pain in teachers at the University of Nairobi and analyzes the relationship between the incidence of low back pain and some variables taken as risk factors.

 In general, the manuscript was well written, and the study was well conducted, with a consistent statistical analysis and a good discussion.

 I only have a few minor comments and suggestions.

**Page 4 - Sample size:**

- Unfortunately, the calculated sample size could not be guaranteed. Given the possibility of sample loss in most studies, why did the authors not start the study with a larger sample than that calculated here?

**Page 4 - Study variables:**

- I think it would be interesting if the authors could justify the choice of the study variables/factors analyzed here. I think some other factors are, in some extension, more important than that cited here.
- For example: office chair design is important, but I think the number of hours of sitting work is more or as important as office chair design. Some comments about this would be welcome. Something like "teachers spend most of their time working in a sitting position, thus we considered the chair design as a risk factor for LBP", for example.
- However, a question can be raised: why not evaluate the number of hours of sitting work?

 

**Conceptual framework in Figure 2:**

- I recommend emphasizing that those relationships presented in Figure 2 are a conception of the authors that motivate the choice of the "risk factors" presented here.
- Those relationships may be different in other conditions, or for other populations, or even other authors may interpret those relationships in a different way.

 

**Page 5 - Figure 2 captions:**

- I recommend not using this term here: "causal diagram". We cannot say that those factors are causal factors of LBP: the cause-effect relationship between them and LBP cannot be guaranteed. Furthermore, the relationships presented here are a personal interpretation of the authors. Other authors may establish a different relationship between the factors presented here. For example: for the authors, the level of stress is a risk factor for LBP, but, perhaps, LBP increases the level of stress, especially for a teacher who works seated most of the time.

 

**Page 6 - 4.2 Risk factors for LBP:**

- I suggest changing this subsection to something such as "Variables studied as risk factors for LBP".
- There are other variables that may be related to LBP, and some variables studied here may not be considered risk factors for LBP by other authors.

 

**Page 7 - Last paragraph:**

- The interpretation here is somewhat speculative. However, the level of physical activity for each age group can be verified. The authors have this information.
- This information would be welcome and would support the interpretation raised in that paragraph.

Is the work clearly and accurately presented and does it cite the current literature?

Yes

If applicable, is the statistical analysis and its interpretation appropriate?

Yes

Are all the source data underlying the results available to ensure full reproducibility?

Yes

Is the study design appropriate and is the work technically sound?

Yes

Are the conclusions drawn adequately supported by the results?

Yes

Are sufficient details of methods and analysis provided to allow replication by others?

Yes

Reviewer Expertise:

Biomechanics, gait and posture, pattern recognition, feature extraction, feature selection, feature classification.

I confirm that I have read this submission and believe that I have an appropriate level of expertise to confirm that it is of an acceptable scientific standard.
